# A meta-analysis of measurement properties of the Western Ontario Meniscal Evaluation Tool (WOMET)

**DOI:** 10.1186/s13018-020-02103-9

**Published:** 2020-11-30

**Authors:** Nikolas Leon Krott, Marcel Betsch, Michael Wild

**Affiliations:** 1 Department of Orthopaedics, University Hispital RWTH Aachen, Pauwelsstraße 30, Aachen, 52074 Germany; 2 Department of Orthopaedics Trauma and Hand Surgery, Klinikum Darmstadt, Darmstadt, Germany

**Keywords:** COSMIN, Meta-analysis, Measurement properties, Patient-reported outcome measures, WOMET, Meniscal pathologies

## Abstract

**Background:**

We provide a meta-analysis for clinicians and researchers regarding the psychometric properties of the WOMET as a patient-reported outcome measure (PROM) for patients with meniscal pathologies.

**Methods:**

A comprehensive literature search identified 6 eligible papers evaluating WOMET measurement properties in patients with different meniscal injuries and meniscal treatments following the Preferred Reporting Items for Systematic Reviews and Meta-Analyses (PRISMA) statement. The quality of the included studies was evaluated using the four-point Consensus-based Standard for the selection of health Measurement Instruments (COSMIN) Checklist for good measurement properties. The checklist was specifically developed for studies on health-related PROMs.

**Results:**

Our meta-analysis suggests that the WOMET can be used to evaluate patients with different meniscal injuries and meniscal treatments, especially acute or chronic meniscal injuries and traumatic or degenerative meniscal injuries treated operatively or conservatively. The WOMET shows satisfactory internal consistency, test-retest reliability, and construct validity. Due to limitations in both sample sizes and methodologies of the included studies, no conclusions can be drawn regarding the WOMET’s content validity, structure validity, cross-cultural validity, measurement error, or responsiveness. A further limitation of the studies included in this meta-analysis is the lack of cross-cultural validation, although recommended by the COSMIN Standards.

**Conclusions:**

The first meta-analysis on measurement properties of the WOMET demonstrates satisfactory internal consistency, test-retest reliability, and construct validity. Further studies are needed, focusing on the methodological deficiencies highlighted in this meta-analysis. To ensure that the WOMET adequately reflects the symptoms, functions, and quality of life of patients with meniscal tears based on COSMIN criteria, it is necessary to assess the structural validity and content validity of this PROM.

## Background

In the diagnosis and treatment of knee pain, patient-reported outcome measures (PROMs) are often used to assess symptoms and effects of therapeutic interventions. PROMs thereby focus on the patients’ evaluation of their status of health. Since PROMs are a central part of medical research and clinical practice, it seems crucial to assess their measurement quality.

Meniscal pathologies are a common knee injury, which can be classified into traumatic or sports-related tears or degenerative knee lesions [1]. The treatment outcomes of knee pathologies have traditionally focused on clinical examination, radiographic imaging, or assessment of range of motion. In recent years, PROMs have gained more importance in evaluating treatment effects, considering patients’ expectations and evaluations of interventions.

One such PROM to evaluate patients with meniscal tears is the Western Ontario Meniscal Evaluation Tool (WOMET). The WOMET was developed by Kirkley et al. in 2007 [2] to assess health-related quality of life (HRQoL) in patients with meniscal tears. The WOMET consists of 16 items along three dimensions (section A: physical symptoms (9 items); section B: sports/recreation/work/lifestyle (4 items); section C: emotions (3 items)). All items are measured and weighed on VAS (visual analog scales); the maximum score is 100, which is converted into a percentage score. Although the WOMET has been extensively used, only a few studies have investigated its psychometric properties.

In the present article, we evaluate the quality of the WOMET, using the COSMIN (Consensus-based Standard for the selection of health Measurement Instruments) Checklist for good measurement properties. The COSMIN is a validated and well-accepted tool to rate the quality of PROMs [3]. Specifically, the COSMIN guidelines have been developed to assist researchers to determine the clinimetric and psychometric soundness of health-related, patient-reported outcomes [3–5]. This is the first meta-analysis that has assessed the psychometric properties of the WOMET as a PROM for patients with meniscal pathologies. By this, we aimed to provide statistical evidence for the quality of the WOMET using pooled data from single studies that investigated the measurement qualities of the WOMET [6].

## Methods

This meta-analysis was performed according to the Preferred Reporting Items for Systematic Reviews and Meta-Analyses (PRISMA) statement [7]. To evaluate measurement properties, we used the COSMIN Standard.

### Literature search

A structured search of the electronic databases MEDLINE via PubMed, EMBASE via OVID, and Cochrane Library was conducted in January 2019 with no language restrictions by two authors (NK and MB). In accordance with COSMIN recommendations, we applied the search filter described by Terwee et al. [8] and a Google Scholar reference check for additional studies. The structured search strategies were designed using the following search terms in the categories: Constructs (HR-PRO or HRPRO or HRQL or HRQoL or QL or QoL or quality of life or health status), Target population (meniscal pathology or meniscal tears or Tibial Meniscus Injuries or Menisci, Tibial), Measurement Instrument (WOMET OR Western Ontario Meniscal Evaluation Tool AND meniscal tears OR meniscal injuries), and Measurement properties (sensitive COSMIN search filter for measurement properties in MEDLINE).

### Eligibility criteria

We included original research articles, systematic reviews, and validation studies with the aim to evaluate at least one measurement property (i.e., reliability, validity, or responsiveness) in accordance with the COSMIN taxonomy [4]. Participants in the selected studies were suffering from any form of meniscal pathologies. Thus, meniscal pathology and WOMET were the constructs of interest for the present meta-analysis (i.e., relevant records). Included articles assessed the measurement properties, development, or interpretability of the WOMET in a majority patient population of adults with meniscus pathologies. The majority is defined as equal to or greater than 50% of the sample.

We excluded studies that evaluated the treatment efficacy without assessing measurement properties and studies in which the WOMET was used to validate another instrument (i.e., inappropriate study designs). Also, studies with less than 50% of patients having a meniscal tear as the primary diagnosis (i.e., without other significant knee pathologies, for example, concomitant anterior cruciate ligament (ACL) rupture) were excluded unless the meniscal tear group was reported separately.

### Article selection

**Fig. 1:**
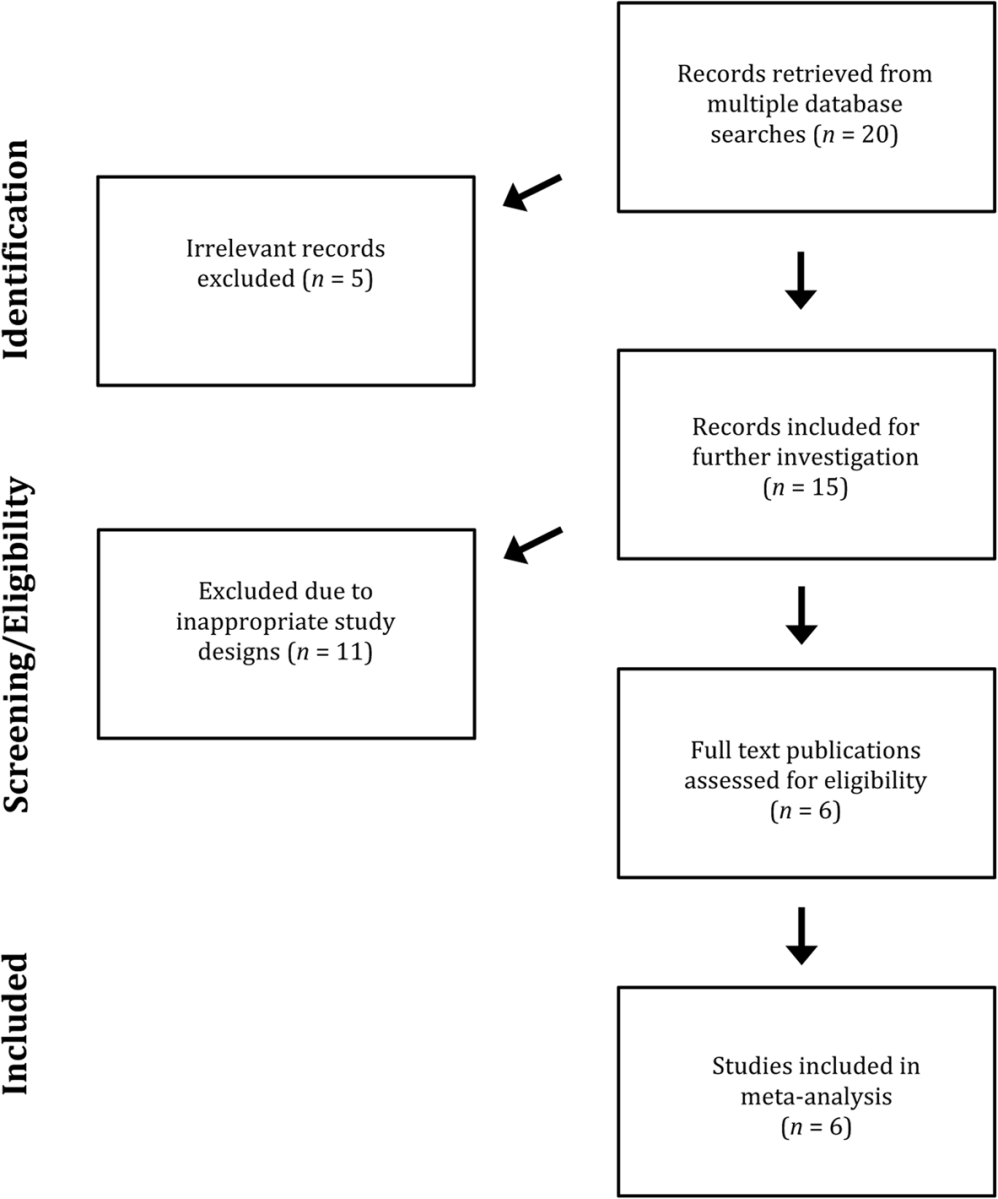
PRISMA flow chart of publication search process

The results of the database searches were examined regarding their titles and abstracts. Then, the selected full-text articles were examined regarding the previously described eligibility criteria (Fig. 1).

### Data extraction

We used spreadsheets to extract sample characteristics and measurement property results for each selected study.

### Evaluation of studies and measurement properties

The quality of the included studies was evaluated by the authors using the four-point COSMIN Checklist for good measurement properties [5, 8]. The checklist was specifically developed for studies on health-related PROMs. It can be used to assess whether a study meets the standard for good methodological quality regarding the following properties: internal consistency, reliability, measurement error, content validity, construct validity, criterion validity, and responsiveness. The checklist is a validated tool comprising 10 sections, with each assessing a separate measurement property [3]. Scoring of single studies follows the concept of “worst score counts” in order to account for poor methodological aspects in the meta-analysis [8]. For example, if for a validation study, one item in the COSMIN check box is rated as “inadequate”, the overall methodological quality of that validation study is rated as “inadequate.” For studies for which quality ratings already existed on the COSMIN website, we adopted those pre-existing scores for our meta-analysis (i.e., the scores obtained in the systematic review by Abram et al. [6]). We also rated the evidence from the studies regarding its quality and bias according to the COSMIN risk of bias checklist (see Appendix, Tables 3 and 4).

### Meta-analytic strategy

We conducted a meta-analysis consulting the MAVIS Meta-Analysis via Shiny software (version 2.1; Hamilton, Aydin, and Mizumoto, 2014 [9]), to assess the general effect sizes of the measurement properties of the WOMET. We used a random effects model to analyze all six studies. The random effects model assumes that the studies in the meta-analysis come from populations with different average effect sizes; that is, population effect sizes can be explained as being sampled from a larger universe of studies [10]. Random-effects models therefore allow to generalize the findings beyond the studies included. We used the random effects model because the studies were drawn from populations that differ from each other in ways that could impact the effects (e.g., patients with traumatic or degenerative meniscal tears, meniscal tears with or without osteoarthritis).

We calculated the effect sizes using Pearson’s correlation coefficient *r*. This coefficient is a standardized form of covariance between two variables and is able to measure the strength of the relationship between continuous variables. After calculating the effect sizes from each study, we tested the heterogeneity of the effect sizes for each measurement property.

## Results

After the literature search, the evaluation of measurement properties, and the quality rating of the six included studies [2, 11–15], we tested the heterogeneity of the effect sizes of the included studies in order to examine the overall effect (see Table 1 for an overview of the psychometric properties reported in the single studies and Table 2 for an overview of the methodological qualities of the single studies).

**Table 1:** Overall ratings of measurement properties and level of evidence (criteria for good measurement properties)

Study	Content validity	Structural validity	Internal consistency	Cross-cultural validity	Reliability	Measurement error	Hypotheses testing for construct validity	Responsiveness
Kirkley et al. [2]	+++	NA	?	NA	+	NA	+	+
Shivonen et al. [11]	?	NA	?	?	?	NA	+	?
Celik et al. [12]	?	NA	?	?	++	?	++	NA
Tong et al. [13]	?	NA	?	?	++	NA	++	?
van der Wal et al. [14]	++	NA	?	?	++	– –	++	++
Sgroi et al. [15]	?	NA	?	?	+	++	+	NA

+++ or – – –, strong evidence positive/negative result

++ or – –, moderate evidence positive/negative result

+ or –, limited evidence positive/negative result

? unknown, due to poor methodological quality

*NA* no information available

**Table 2:** Quality of each study (COSMIN-rating)

Study	Content validity	Structural validity	Internal consistency	Cross-cultural validity	Reliability	Measurement error	Hypotheses testing for construct validity	Responsiveness
Kirkley et al. [2]	Excellent	na	Poor	na	Fair	na	Fair	Fair
Shivonen et al. [11]	Poor	na	Poor	Poor	Poor	na	Fair	Poor
Celik et al. [12]	Poor	na	Poor	Poor	Good	Good	Good	na
Tong et al. [13]	Poor	na	Poor	Poor	Good	na	Good	Poor
van der Wal et al. [14]	Good	na	Poor	Poor	Good	Good	Good	Good
Sgroi et al. [15]	Poor	na	Poor	Poor	Fair	Good	Fair	na

### Internal consistency

**Fig. 2:**
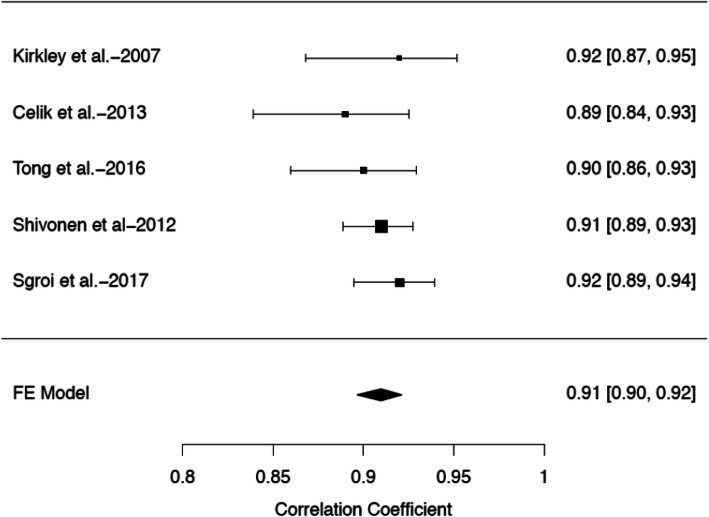
Forest plot, random effects model for the internal consistency of the WOMET

The test for heterogeneity revealed that the effect sizes for the measurement property internal consistency did not significantly differ between the five studies included, *p* = 0.677, indicating homogeneous effects of the five studies (*H* = 1.00, 95% CI [1.00, 1.67], *I*^2^ = 0%). We excluded the study by van der Wal et al. [14], because it did not report overall internal consistency of the WOMET, but instead reported internal consistency of its subscales. The estimated overall correlation coefficient was *r* = 0.91, 95% CI [0.90, 0.92], *z* = 42.20 *p* < 0.001 (see Fig. 2 in Appendix). The test revealed no indication for publication bias, with 3005 non-significant studies necessary to make the result non-significant.

### Test-retest reliability

**Fig. 3:**
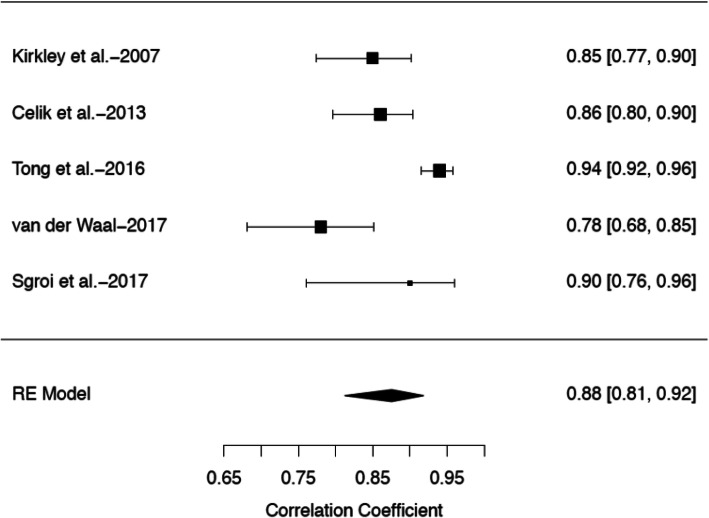
Forest plot, random effects model for the Test-retest reliability of the WOMET

The test for heterogeneity revealed that the effect sizes for the measurement property test-retest reliability did significantly differ between the five studies included, *p* < 0.001, indicating heterogeneous effects of the five studies (*H* = 2.57, 95% CI [1.72, 3.83], *I*^2^ = 84%). The estimated overall correlation coefficient was *r* = 0.88, 95% CI [0.80, 0.93], *z* = 9.90 *p* = 0.001 (see Fig. 3 in Appendix). The test revealed no indication for publication bias, with 1231 non-significant studies necessary to make the result non-significant.

### Construct validity

The included studies evaluated construct validity using different scales. The studies by Celik [12] and Tong [13] tested the WOMET against different subscales of the short-form health survey 36 (SF-36) [16]. Kirkley [2], Celik [12], and Shivonen [11] tested the WOMET against the Lysholm Scale [17], and Tong [13] and van der Waal [14] used the International Knee Documentation Committee Subjective Knee Form (IKDC) [18] to assess the WOMET’s construct validity.

### SF-36

The following subscales from the SF-36 were investigated: physical functioning, bodily pain, role-emotional, and mental health.

**Fig. 4:**
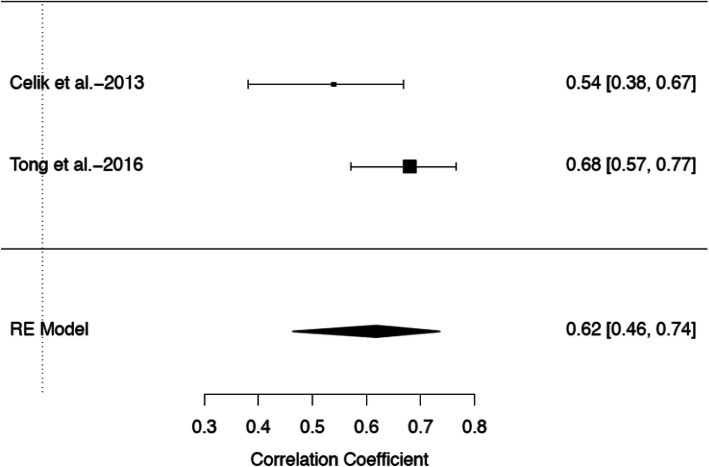
Forest plot, random effects model for the construct validity of the SF-36 PF and WOMET

**Fig. 5:**
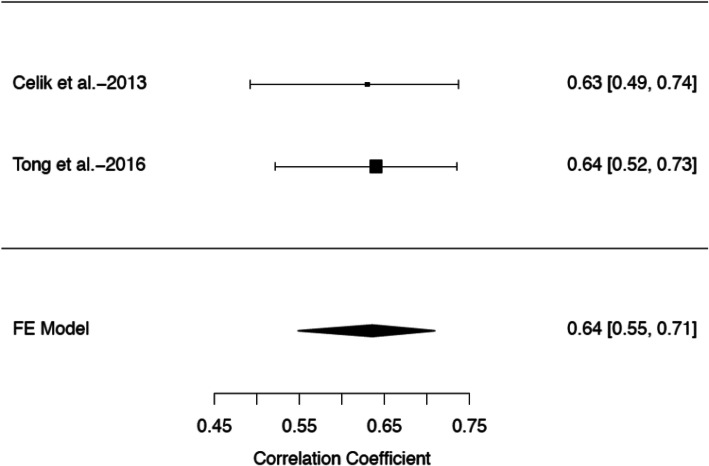
Forest plot, random effects model for the construct validity of the SF-36 BP and WOMET

**Fig. 6:**
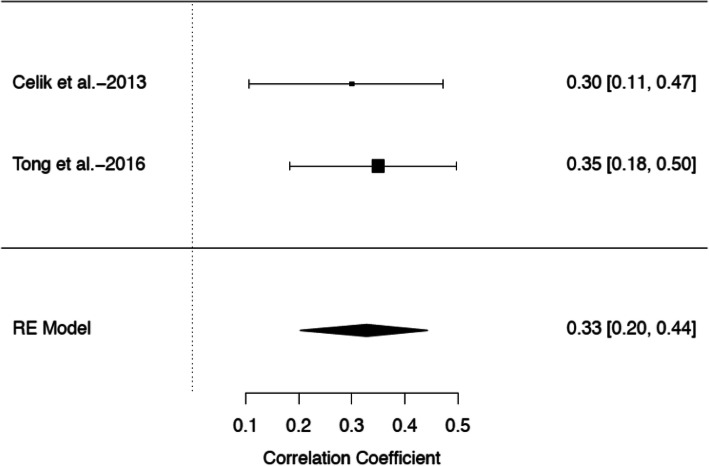
Forest plot, random effects model for the construct validity of the SF-36 MH and WOMET

For the subscales physical functioning, bodily pain, and mental health, the test for heterogeneity revealed that the effect sizes for construct validity did not significantly differ between the two studies, *p*s > 0.103, indicating homogeneous effects of the two studies (physical functioning: *H* = 1.62, *I*^2^ = 62%; bodily pain: *H* = 1.00, *I*^2^ = 0%; mental health: *H* = 1.00, *I*^2^ = 0%). The estimated overall correlation coefficients were moderate to high (physical functioning: *r* = 0.62, 95% CI [0.46, 0.74], *z* = 6.42, *p* = 0.001; bodily pain: *r* = 0.64, 95% CI [0.55, 0.71], *z* = 10.91 *p* = 0.001; mental health: *r* = 0.33, 95% CI [0.20, 0.44], *z* = 4.95 *p* = 0.001; see Figs. 4, 5, and 6 in Appendix). The tests revealed no indication for publication bias, with a moderate to high number of non-significant studies necessary to make the result non-significant (physical functioning *n* = 80; bodily pain *n* = 86; mental health *n* = 16).

**Fig. 7:**
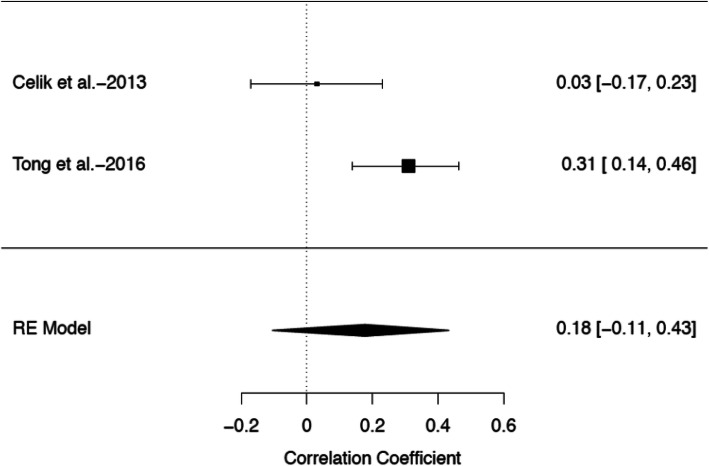
Forest plot, random effects model for the construct validity of the SF-36 RE and WOMET

For the subscale role-emotional, the test for heterogeneity revealed that the effect sizes for construct validity did significantly differ between the two studies, *p* = 0.036, indicating heterogeneous effects of the two studies (*H* = 2.10, *I*^2^ = 77%). The estimated overall correlation coefficient is *r* = 0.18, 95% CI [− 0.11, 0.43], *z* = 1.23 *p* = 0.217 (see Fig. 7 in Appendix). There was no indication for publication bias (*n* = 4 studies necessary for non-significant results).

### Lysholm Score

**Fig. 8:**
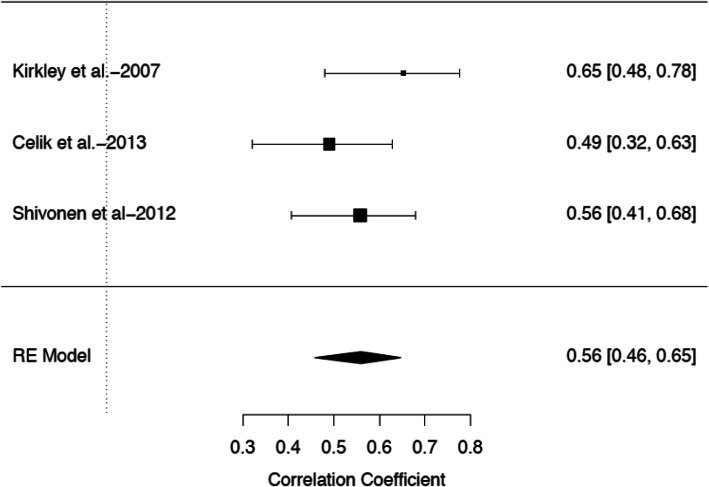
Forest plot, random effects model for the construct validity of the Lysholm Scale and WOMET

For the Lysholm Score, the test for heterogeneity revealed that the effect sizes for construct validity did significantly differ between the three studies *p* = 0.343, indicating heterogeneous effects of the two studies (*H* = 1.03, 95% CI [1.00, 3.04], *I*^2^ = 6.3%). The estimated overall correlation coefficient was *r* = 0.56, 95% CI [0.46, 0.64], *z* = 9.60 *p* = 0.001 (see Fig. 8 in Appendix). The test revealed no indication for publication bias, with 108 non-significant studies necessary to make the result non-significant.

### IKDC

**Fig. 9:**
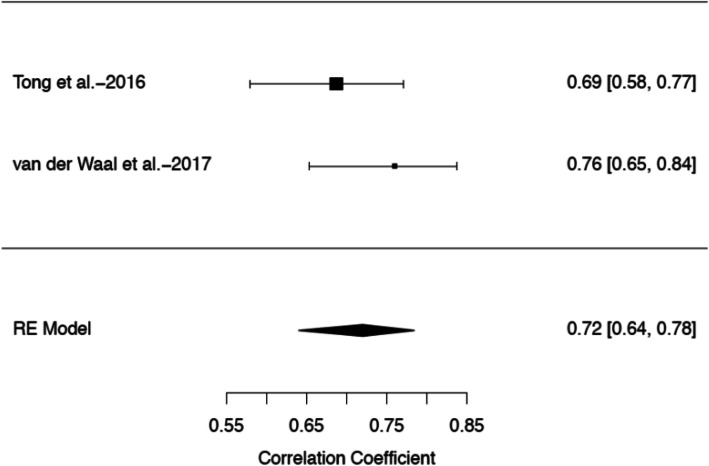
Forest plot, random effects model for the construct validity of the IKDC and WOMET

For the IKDC, the test for heterogeneity revealed that the effect sizes for the construct validity did not significantly differ between the two studies, *p* = 0.300, indicating homogeneous effects of the two studies (*H* = 1.03, *I*^2^ = 6.6%). The estimated overall correlation coefficient was *r* = 0.72, 95% CI [0.65, 0.78], *z* = 12.44 *p* = 0.001 (see Fig. 9 in Appendix). There further was no indication for publication bias (*n* = 122 studies necessary for non-significant results).

Overall, using pooled data from multiple studies, our results show that the WOMET shows high internal consistency, with values ranging from 0.90 to 0.92. Further, when administered multiple times, scores of the WOMET were reliable across studies (*r* = 0.80–0.93). Lastly, the WOMET showed high construct validity when compared with other scales such as the SF-36, the Lysholm Score, or the IKDC.

## Discussion

By means of this meta-analysis, we combined previous results within a new statistical framework. The results suggest that the WOMET can be used as a patient-reported outcome measure for the evaluation of patients with different meniscal injuries. Our paper is the first to present statistical evidence on the psychometric properties of the WOMET. The WOMET showed good psychometric properties across the studies included and the meta-analytic results suggest that effect sizes are not due to publication or selection bias. The WOMET shows satisfactory internal consistency, test-retest reliability, and construct validity. Due to limitations in both sample sizes and methodologies of the included studies, no conclusions can be drawn regarding the WOMET’s content validity, structure validity, cross-cultural validity, measurement error, or responsiveness. A further limitation of the studies included in this meta-analysis is the lack of cross-cultural validation, although recommended by the COSMIN Standards [3]. A PROM is seen as cross-culturally valid if, for example, a Dutch and an English version are comparable in that they obtain similar results in a comparable population. Future studies should evaluate existing and new language versions of the WOMET regarding their cross-cultural validity.

Abram et al. [6] published a systematic review to evaluate PROMs for patients with meniscal pathologies. They found that the Lysholm Score [17], the IKDC [18], and the KOOS (Knee Osteoarthritis Score) [19] showed limited ability to assess symptoms and functional status in patients with meniscal tears. In contrast, they found that the WOMET had high content validity. In accordance with the results obtained by Abram et al. [6], our meta-analysis provides the first statistical evidence to recommend the WOMET as an instrument to evaluate treatment effects in patients with different meniscal injuries and meniscal treatments, especially acute or chronic meniscal injuries and traumatic or degenerative meniscal injuries treated operatively or conservatively. Whereas Abram et al. qualitatively summarized results from previous studies on measurement properties of the WOMET, we performed quantitative analyses on statistically pooled data from multiple studies in order to draw conclusions about overall effects.

Compared to other instruments, such as the IKDC or the Lysholm Score, the WOMET shows superior content validity, because it is the only measurement instrument which was developed based on patient rating regarding its comprehensiveness, feasibility, and comprehensibility. In contrast, the Lysholm Score was developed as a disease-specific measurement for patients suffering from knee ligament injuries, whereas the IKDC score was developed as a global knee-specific measurement. One study suggests that measurement error may limit the ability of the WOMET to detect the MIC in score for meniscal patients [14]. Generally, strong methodological evaluations of the structural validity of many PROMs are still lacking. When conducting a systematic review or a meta-analysis, the lack of studies that report methodological details is an essential problem, because it limits the possibility to assess the methodological quality of the studies. In the areas of randomized controlled trials or diagnostic research, there exist guidelines for primary studies, such as the Consolidated Standards of Reporting Trials (CONSORT) statement [20] or the Standards for Reporting of Diagnostic accuracy studies (STARD) statement [21]. In research on measurement properties, however, there exist wide variations in names given to specific measurement properties and different definitions are used for the same property. For example, the measurement of property reliability has also been referred to as reproducibility or stability [22]. We therefore use and recommend the COSMIN checklist [3] as an adequate guideline on evaluating measurement properties.

## Limitations

For most of the studies included in this meta-analysis, the COSMIN methodology rating was poor for the reported measurement properties. Internal consistency was even rated poor in all studies. A key reason for this is the failure of most studies to analyze the factor structure of the WOMET. Without the assessment of the factor structure, there is no possibility of a clear interpretation of internal consistency (see also Tables 3 and 4 in the Appendix). The same holds for the interpretation of change scores: future studies should assess change scores of the WOMET in individual patients. Further, potential publication bias should be taken into account when considering the results of the present meta-analysis.

## Conclusions

This is the first meta-analysis on measurement properties of the WOMET. We found satisfactory internal consistency, test-retest reliability, and construct validity.

Due to the lack of methodological quality as recommended by the COSMIN standards, we are unable to report structure validity or content validity of the WOMET. To ensure that the WOMET adequately reflects the symptoms, functions, and quality of life of patients with meniscal tears based on COSMIN criteria, it is necessary to assess the structural validity and content validity of this PROM if further studies.

## Data Availability

Since no original data were collected for this research, we did not store the data in a repository. Any materials and data of the present meta-analysis are available on request from the first author.

## Appendix

**Table 3:** COSMIN risk of bias checklist

PROM (ref)	Distribution of scores in the study population	Percentage of missing items and missing total scores	Floor and ceiling effects	Minimal important change (MIC) or minimal important difference (MID)
**WOMET (Kirkley et al.** [2]**)**	Not reported	Not reported	5.7% ceiling 1.7% floor	Not reported
**WOMET (Shivonen et al.** [11]**)**	*M* = 47 SD = 17	**0%**	0% ceiling 0% floor	Not reported
**WOMET (Celik et al.** [12]**)**	*M* = 1048,9 SD = 271,6	**0%**	0% ceiling 0% floor	MID 103.2
**WOMET (Tong et al.** [13]**)**	*M* = 71,6 SD = 20.1	**0%**	0% ceiling 0% floor	Not reported
**WOMET (van der Wal et al.** [14]**)**	*M* = 40.8 SD = 15.8	**1.18%**	< 15% ceiling < 15% floor	MIC 14.7
**WOMET (Sgroi et al.** [15]**)**	*M* = 52.0 SD = 15.5	Not reported	0% ceiling 0% floor	MID 12.7

**Table 4:** COSMIN standard summary of findings for each study

PROM (ref)	Summary	Overall rating	Quality of evidence
**Structural validity**			
WOMET (Kirkley et al. [2])		**No info available**	
WOMET (Shivonen et al. [11])		**No info available**	
WOMET (Celik et al. [12])		**No info available**	
WOMET (Tong et al. [13])		**No info available**	
WOMET (van der Wal et al. [14])		**No info available**	
WOMET (Sgroi et al. [15])		**No info available**	
**Internal consistency**			
WOMET (Kirkley et al. [2])	Cronbach’s *α* = 0.92	**Sufficient**	**Low**
WOMET (Shivonen et al. [11])	Cronbach’s *α* = 0.75–0.92	**Insufficient**	**Low**
WOMET (Celik et al. [12])	Cronbach’s *α* = 0.89	**Sufficient**	**Low**
WOMET (Tong et al. [13])	Cronbach’s *α* = 0.90	**Insufficient**	**Low**
WOMET (van der Wal et al. [14])	Cronbach’s *α* = 0.79–0.87	**Sufficient**	**Low**
WOMET (Sgroi et al. [15])	Cronbach’s *α* = 0.92	**Insufficient**	**Low**
**Cross-cultural validity**			
WOMET (Kirkley et al. [2])		**No info available**	
WOMET (Shivonen et al. [11])		**No info available**	
WOMET (Celik et al. [12])		**insufficient**	**Low**
WOMET (Tong et al. [13])		**No info available**	
WOMET (van der Wal et al. [14])		**No info available**	
WOMET (Sgroi et al. [15])		**No info available**	
**Reliability**			
WOMET (Kirkley et al. [2])	Test-retest ICC = 0.73–0.87	**Sufficient**	**High**
WOMET (Shivonen et al. [11])		**No info available**	
WOMET (Celik et al. [12])	Test-retest ICC = 0.86		**Moderate**
WOMET (Tong et al. [13])	Test-retest ICC = 0.94	**Sufficient**	**Moderate**
WOMET (van der Wal et al. [14])	Test-retest ICC = 0.78	**Sufficient**	**Moderate**
WOMET (Sgroi et al. [15])	Test-retest ICC = 0.90	**Sufficient**	**High**
**Measurement error**			
WOMET (Kirkley et al. [2])		**No info available**	
WOMET (Shivonen et al. [11])		**No info available**	
WOMET (Celik et al. [12])	SEM = 32.7; MDC = 103.2	**Insufficient**	**Moderate**
WOMET (Tong et al. [13])		**No info available**	
WOMET (van der Wal et al. [14])	SDC = 20.5; MIC = 14.7	**Insufficient**	**High**
WOMET (Sgroi et al. [15])	SEM = 4.6; MDC = 12.7	**Insufficient**	**Moderate**
**Hypotheses testing**			
WOMET (Kirkley et al. [2])	Predefined hypotheses were confirmed	**Sufficient**	**Moderate**
WOMET (Shivonen et al. [11])	Hypotheses were significant	**Sufficient**	**Low**
WOMET (Celik et al. [12])		**No info available**	
WOMET (Tong et al. [13])	Hypotheses were significant	**Sufficient**	**Low**
WOMET (van der Wal et al. [14])	11 out 14 hypotheses confirmed	**Sufficient**	**High**
WOMET (Sgroi et al. [15])	Predefined hypotheses were confirmed	**Sufficient**	**Moderate**
**Responsiveness**			
WOMET (Kirkley et al. [2])	SRM = 0.65	**Insufficient**	**Low**
WOMET (Shivonen et al. [11])	SRM = 0.90	**Insufficient**	**Low**
WOMET (Celik et al. [12])		**No info available**	
WOMET (Tong et al. [13])	SRM =1.11	**Insufficient**	**Low**
WOMET (van der Wal et al. [14])	*r* = 0.78	**Sufficient**	**High**
WOMET (Sgroi et al. [15])		**No info available**	

## Abbreviations

PROM: Patient-reported outcome measures
WOMET: Western Ontario Meniscal Evaluation Tool
HRQoL: Health-related quality of life
COSMIN: Consensus-based Standard for the selection of health Measurement Instruments
PRISMA: Preferred Reporting Items for Systematic Reviews and Meta-Analyses
SF-36: Short-form health survey 36
IKDC: International Knee Documentation Committee Subjective Knee Form
KOOS: Knee Osteoarthritis Score
CONSORT: Consolidated Standards of Reporting Trials
STARD: Standards for Reporting of Diagnostic accuracy studies
